# Aggregation of Gold Nanoparticles Caused in Two Different Ways Involved in 4-Mercaptophenylboronic Acidand Hydrogen Peroxide

**DOI:** 10.3390/ma12111802

**Published:** 2019-06-03

**Authors:** Runmei Li, Xuefan Gu, Xingtang Liang, Shi Hou, Daodao Hu

**Affiliations:** 1 Engineering Research Center of Historical and Cultural Heritage Protection, Ministry of Education, School of Materials Science and Engineering, Shaanxi Normal University, Xi’an 710062, China; lrm@snnu.edu.cn (R.L.); qzxylxt@163.com (X.L.); houshi@snnu.edu.cn (S.H.); 2 College of Chemistry and Chemical Engineering, Xi’an Shiyou University, Xi’an 710065, China; XuefanGu@xsyu.edu.cn

**Keywords:** 4-mercaptophenylboronic acid, AuNPs, H_2_O_2_, aggregation, bis(4-hydroxyphenyl) disulfide, peroxoboric acid

## Abstract

The difference in gold nanoparticle (AuNPs) aggregation caused by different mixing orders of AuNPs, 4-mercaptophenylboronic acid (4-MPBA), and hydrogen peroxide (H_2_O_2_) has been scarcely reported. We have found that the color change of a ((4-MPBA + AuNPs) + H_2_O_2_) mixture caused by H_2_O_2_ is more sensitive than that of a ((4-MPBA + H_2_O_2_) + AuNPs) mixture. For the former mixture, the color changes obviously with H_2_O_2_ concentrations in the range of 0~0.025%. However, for the latter mixture, the corresponding H_2_O_2_ concentration is in the range of 0~1.93%. The mechanisms on the color change originating from the aggregation of AuNPs occurring in the two mixtures were investigated in detail. For the ((4-MPBA + H_2_O_2_) + AuNPs) mixture, free 4-MPBA is oxidized by H_2_O_2_ to form bis(4-hydroxyphenyl) disulfide (BHPD) and peroxoboric acid. However, for the ((4-MPBA+AuNPs) + H_2_O_2_) mixture, immobilized 4-MPBA is oxidized by H_2_O_2_ to form 4-hydroxythiophenol (4-HTP) and boric acid. The decrease in charge on the surface of AuNPs caused by BHPD, which has alarger steric hindrance, is poorer than that caused by -4-HTP, and this is mainly responsible for the difference in the aggregation of AuNPs in the two mixtures. The formation of boric acid and peroxoboric acid in the reaction between 4-MPBA and H_2_O_2_ can alter the pH of the medium, and the effect of the pH change on the aggregation of AuNPs should not be ignored. These findings not only offer a new strategy in colorimetric assays to expand the detection range of hydrogen peroxide concentrations but also assist in deepening the understanding of the aggregation of citrate-capped AuNPs involved in 4-MPBA and H_2_O_2_, as well as in developing other probes.

## 1. Introduction

Gold nanoparticle (AuNP) probes based on colorimetric strategy have drawn increasing attention due to their simplicity, high sensitivity, and low cost [1]. A well-dispersed AuNP colloid dispersion exhibits a red color; a solution of aggregated AuNPs, however, appears purple or blue. The color change of AuNPs is related to the distance-dependent plasmon coupling of the inter-particles [2]. Based on this principle, many of AuNP-based colorimetric assays for the detection of various analytes have been reported. These colorimetric assays are established on the change of the dispersion behavior of AuNP striggered by target analytes. Functional molecules used to modify AuNPs are the key to finding various specific analytical methods. The combination of boronic acids specially reacted with 1,2- or 1,3-diols [3,4,5,6] and thiol specially tethered onto the surface of AuNPs, 4-mercaptophenylboronic acid(4-MPBA), is one of the desirable candidates that has been widely employed. By employing 4-MPBA-modified AuNPs (4-MPBA/AuNPs), various AuNP-based colorimetric assays have been constructed. For these methods, the selective reactions of the thiol group or boronic acid group on the surface of 4-MPBA-modified AuNPs with the target analytes can trigger the aggregation or dis-aggregation of AuNPs with an accompanying color change. Based on this analytic platform, the colorimetric methods have been developed to detect target analytes including sugars [7], sialic acid [8], catechol [9], Hg^2+^ [10,11], ATP [12], and dopamine [13].

For these methods, some probes are based on the reaction of4-MPBA/AuNPs with H_2_O_2_. For these probes, the selective and efficient reaction between the boronic acid group of 4-MPBA and H_2_O_2_ can form 4-hydroxythiophenol (4-HTP) [14,15,16], which induces the change of the AuNP dispersion state due to the change in the components of 4-MPBA/AuNPs or analyte-modified 4-MPBA/AuNPs. By employing this principle, various chemical probes have been widely developed. For instance, in the detection of prostate-specific antigen (PSA), the aggregation of benzene-1,4-diboronic acid (BDBA)-modified AuNPs can be destroyed by H_2_O_2_produced from the oxidation of glucose by the glucose oxidase immobilized by PSA. Based on the aforementioned blue-to-red color change of AuNPs, PSA determination could be achieved [17]. Based on the fact that the reaction between BDBA and H_2_O_2_ forms p-benzenediol to result in the dis-aggregation of aggregated BDBA/AuNPs, the electrochemical and colorimetric assays of hydrogen peroxide could also be constructed [18]. By employing the reaction of H_2_O_2_ with 4-MPBA adsorbed on the surface of a gold mirror, H_2_O_2_ or the glucose selectively bound to 4-MPBA could be detected using surface-enhanced Raman spectroscopy (SERS) [14]. Similarly, 3-mercaptophenylboronic acid (3-MPBA)-modified AuNPs were also used to probe exogenous and endogenous H_2_O_2_ and be coupled with glucose oxidase (GOx) to achieve the selective detection of glucose [16]. It was found that the arylboronate immobilized onto an electrode surface reacts with H_2_O_2_ to yield a change of pH in a range of 2 to 6, which results in a steady-state change in the electrochemical potential. Based on this finding, a potentiometric hydrogen peroxide probe was constructed [18]. Evidently, the combination of 4-MPBA with AuNPs could be constructed into a platform with the versatility to detect various analytes. In these involved approaches, the colorimetric methods have advantages in their simplicity. For instance, detection is possible with the naked eye or UV/Vis spectrometry without requirements for complicated instrumentation or much knowledge involved in the electrochemical or fluorescent systems. Although the colorimetric assays related to 4-MPBA/AuNPs containing H_2_O_2_ have been widely investigated, the difference in the aggregation that occurs in ((4-MPBA + AuNPs) + H_2_O_2_) mixtures and ((H_2_O_2_ + 4-MPBA) + AuNPs) mixtures has so far not been reported.

Interestingly, for the two mixtures, we found that there is a great difference in the concentration range of H_2_O_2_ in inducing AuNP aggregation. Herein, we describe not only the two different approaches to induce aggregation of AuNPs involved in 4-MPBA and H_2_O_2_ but also, especially, their corresponding mechanisms. The results indicate that the difference in the concentration range of hydrogen peroxide in AuNP aggregation is mainly related to the difference in the reaction of H_2_O_2_ with free and immobilized 4-MPBA. In the mixture of ((4-MPBA + AuNPs) + H_2_O_2_), the immobilized 4-MPBAis oxidized by H_2_O_2_ to form 4-hydroxythiophenol (4-HTP). The change of 4-MPBA to 4-HTP makes the loaded charge on the surface of AuNPs decrease, resulting in the aggregation of AuNPs. In the mixture of ((H_2_O_2_ + 4-MPBA) + AuNPs), however, the free 4-MPBA is oxidized by H_2_O_2_ to form bis(4-hydroxyphenyl) disulfide (BHPD), and the immobilization of BHPD with bigger steric hindrance onto surface of AuNPs is more difficult than that of 4-HTP. This difference makes the mixture of ((4-MPBA + AuNPs) + H_2_O_2_ more sensitive to the aggregation of AuNPs caused by H_2_O_2_ compared with the mixture of ((H_2_O_2_ + 4-MPBA) + AuNPs). In addition, some details in the change of pH during the reaction for the two mixtures have also been revealed. The new findings should assist in expanding the detection range of H_2_O_2_ concentrations by AuNP-based colorimetric assays. Additionally, the revealed mechanisms should not only assistin deepening the understanding of the physical and chemical behavior of the system containing AuNPs, 4-MPBA, and H_2_O_2_, but also have a significant positive effect on developing new AuNP-based probes.

## 2. Experimental

### 2.1. Materials

4-Mercaptophenylboronic acid (4-MPBA, purity ≥ 90%) and 4-hydroxythiophenol (4-HTP) were purchased from Sigma Aldrich (Shanghai, China). Tetrachloroauric (HAuCl_4_·4H_2_O, purity ≥ 99.9%), hydrogen peroxide (H_2_O_2_, ≥30%), and absolute ethanol (purity ≥99.99%) were obtained from Sinopharm Chemical Reagent Co., Ltd. (Shanghai, China). Trisodium citrate (AR) and potassium hydroxide (KOH, AR) were received from TIANLI Chemical Reagents Ltd. (Tianjin, China). All reactants were used without further purification. Milli-Q water was used in all experiments. All used glassware was treated with aqua regia, rinsed in Milli-Q water, and oven-dried prior to use.

### 2.2. Synthesis of Gold Nanoparticles

The citrate-stabled AuNPs were synthesized via previously reported methods [19]. Briefly, 1 mL HAuCl_4_ (25 mM) aqueous was injected into a boiling solution of 150 mL sodium citrate (2.2 mM), and then the solution was kept boiling for 10 min. The color of the solution changed from yellow to bluish gray and then to soft pink. After the solution was cooled down to 90 °C, a 2 mL solution was drawn, and then the rest of solution was used as the seed solution (∼10 nm, ∼3 × 10^12^ NPs/mL). Then, 1mL HAuCl_4_ solution(25 mM) was added into the seed solution and maintained at a temperature of 90 °C for 30 min, and this process was repeated twice. After the reaction was quenched by an ice-water bath, AuNPs with ∼20 nm were obtained. The concentration of the final AuNPs was approximately the same as the seed particles (∼3 × 10^12^ NPs/mL). 

### 2.3. Reaction Conditions Involved in the Investigation

To investigate the interactions among 4-MPBA, H_2_O_2_, and AuNPs, some related reactive conditions were selected, as shown in Table 1, and the corresponding samples were used for the subsequent determination or characterization studies.

### 2.4. Characterization Techniques

UV-visible spectra were acquired with a Lambda 35 UV-Vis Spectrometer (PerkinElmer, Waltham, MS, USA). The ^13^C, ^1^H, and ^11^B NMR (nuclear magnetic resonance) spectra were monitored by a JNE-ECZ400S/L1 Nuclear Magnetic Resonance Spectrometer (400 MHz NMR, JEOL, Tokyo, Japan). Raman measurements were performed on an in Via Raman microspectrometer (Renishaw, London, UK) with 532 nm laser excitation. Transmission electron microscopy (TEM) images were recorded on a JEM-2100 system (JEOL, Tokyo, Japan) with an accelerating voltage of 200 kV. The effective surface charge on4-MPBA/AuNPs in the different conditions shown in Table 1 was measured using the Zeta-potential (Malvern Instruments Zetasizer, Worcestershire, UK). X-ray photoelectron spectroscopy (XPS) measurements were performed by X-ray photoelectron spectroscopy (XPS, AXIS ULTRA, Kratos Analytical Ltd., Manchester, UK) using amonochromated Al K_α_ X-ray source (1486.71 eV of photons). Further details can be found in the Supplementary Materials.

## 3. Results and Discussion

### 3.1. The UV-Vis Spectroscopy for the Interaction between 4-MPBA and the Citrate-Capped AuNPs

**Figure 1:**
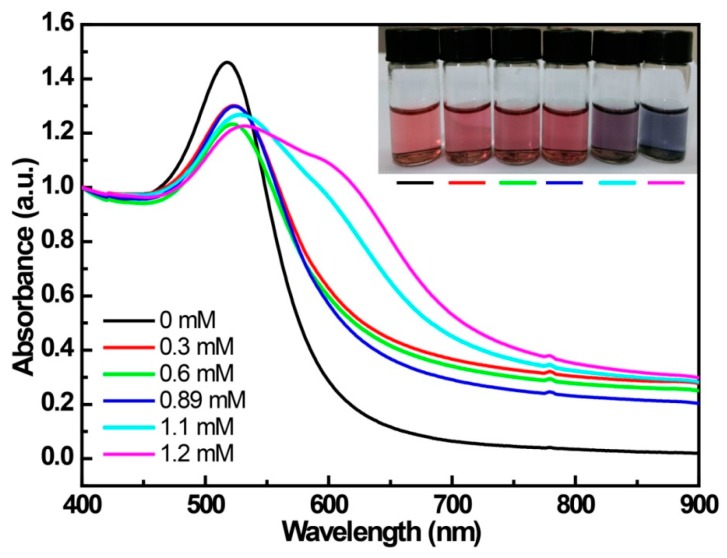
The change in the UV-Vis spectrum of citrate-capped gold nanoparticles (AuNPs) with the addition of different amounts of 4-mercaptophenylboronic acid (4-MPBA).

**Figure 2:**
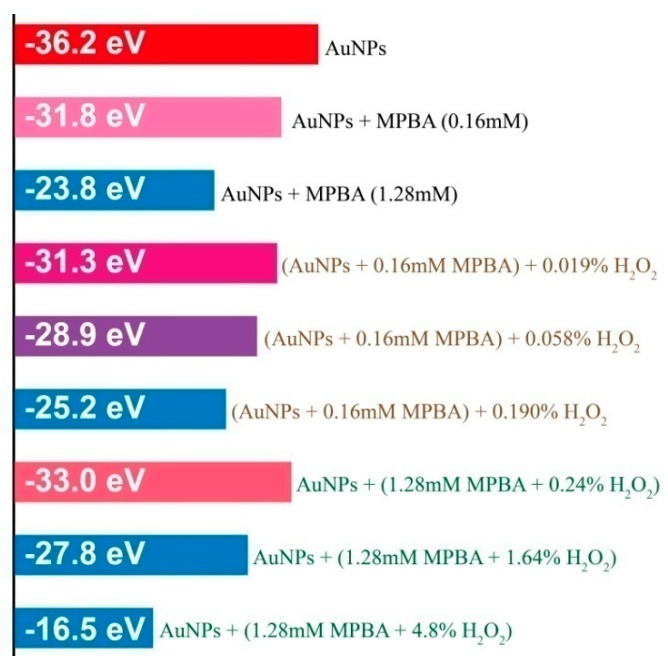
Zeta-potentials for the solutions formed by a given amount of citrate-capped AuNPs mixed with different amounts of 4-MPBA and H_2_O_2_.

The change in UV-Vis spectra of citrate-capped AuNPs with the change in concentration of 4-MPBA is shown in Figure 1. For the prepared citrate-stabilized AuNP solution, a red color, displaying a SPR (surface plasmon resonance) peak at 523 nm (Figure 1, black curve), is exhibited due to the electrostatic repulsion that is derived from the negative charge of the citric acid on the surface between adjacent particles. When the concentration of 4-MPBA increases in the citrate-stabilized AuNP solution, this peak is slightly red-shifted, suggesting the formation of a corona on the AuNP surface caused by the displacement of weakly-bound citrate ions with 4-MPBA [20]. Additionally, the increased amount of 4-MPBA leads to the pronounced appearance of a new absorbance ranging from 525 nm to 800 nm, indicating that the aggregation of AuNPs is enhanced. We speculate that the aggregation is attributed to the decrease in electrostatic interaction. Since citric acid, with three negative charges, is displaced by 4-MPBA, which is loaded with one negative charge at pH above 9.2 (pKa of 4-MPBA) [21], the aggregation of AuNPs should be attributed to the reduction in the negative charge density on the surface of AuNPs, and this tendency continuously strengthens with the increase of 4-MPBA. This deduction was confirmed by the results shown in Figure 2. The absolute value of the Zeta-potential of 4-MPBA/AuNPs reliably decreases with the increase of 4-MPBA, for example: AuNPs (−36.2 eV), AuNPs + 0.16 mM 4-MPBA (−31.8 eV), and AuNPs + 1.28 mM 4-MPBA (−23.8 eV). The aggregation of AuNPs induced by 4-MPBA being attributed to electrostatic interaction was also testified by the following experiments.

Figure 1 shows that the citrate-capped AuNPs are relatively stable in the presence of 4-MPBA concentrations ranging from 0 to 0.89 mM. Figure S1 shows the change in the UV-Vis spectra of 4-MPBA (0.89 mM)-modified citrate-capped AuNPs in which the pH was adjusted by different solutions (including different concentrations of PBS (phospate buffer saline) buffer solutions with different pH values—solutionscontaining HCl or NaOH).For the solutions of AuNPs/4-MPBA with different pH values adjusted by PBS buffer solutions, the absorbance ranging from 525 nm to 800 nm gradually increases with the increase of PBS concentrations and with the decrease of pH values (Figure S1A–D). However, the 4-MPBA/AuNPs solution is stable while the pH is adjusted from 4 to 10 with KOH, HCl, or 5 mM PBS buffer solutions. All above results indicate that the aggregation of 4-MPBA/AuNPs is sensitive to the ionic strength of salts [22,23]. The salt-induced aggregation of AuNPs is considered to be a consequence of the suppressive effect of the salt against the Coulombic repulsive interaction between the citrate-stabilized AuNPs [24]. Additionally, the aggregation of 4-MPBA/AuNPs enhanced with the decrease of pH is also attributed to phosphate protonation, resulting in decrease of the charged number of the phosphate anion. In fact, not surprisingly, the aggregation of 4-MPBA/AuNPs often appears in the solution containing a salt buffer [10,12,25,26,27], and the stabilized 4-MPBA/AuNP solution is usually found in the case of KOH-adjusted pH values [8] or zwitterionic buffers with lower ionic strength (e.g., Tricine and Hepes) [28,29]. Thus, AuNP-based sensing strategies should pay special attention to the dispersion states of AuNPs affected by salt, DNA, proteins, or other ions [23]. 

Combining these reports in the literature with our experimental results, we believe that the aggregation of 4-MPBA/AuNPs is mainly attributed to the decrease of electrostatic repulsive force. In particular, we found that the mixture of the well-dispersed citric acid-capped AuNPs and well-dispersed 4-MPBA/AuNPs also remains well dispersed for a long time, further implying that the reaction between 4-MPBA and citric acid scarcely occurred. This conclusion, i.e., that the aggregation of 4-MPBA/AuNPs is actually attributed to the weakened electrostatic interaction, will be proved again in following experiments. 

### 3.2. The UV-Vis Spectroscopy of AuNP Solutions Containing 4-MPBA and H_2_O_2_

**Figure 3:**
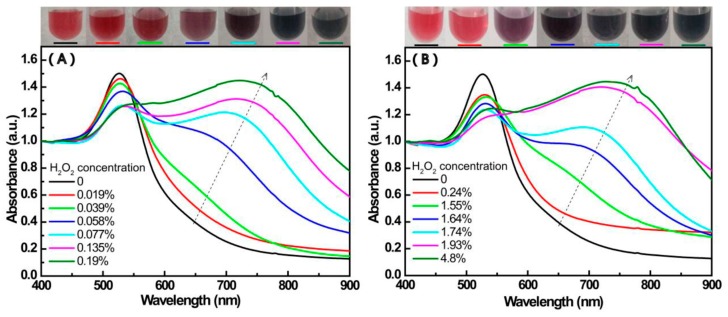
The UV-Vis spectrum of Mixt.1 (**A**) (0.16 mM 4-MPBA) and Mixt.2 (**B**) (1.28 mM) in the presence of different concentrations of H_2_O_2_with photographs of their corresponding samples.

Figure 3 shows the UV-Vis spectra of Mixt.1 (a higher ratio of 4-MPBA to H_2_O_2_) and Mixt.2 (a lower ratio of 4-MPBA to H_2_O_2_).Mixt.1 is a series of mixtures where the given amounts of 4-MPBA and AuNPs were first mixed then followed by the addition of different amounts of H_2_O_2_, while Mixt.2 is a series of mixtures that given amounts of 4-MPBA was first mixed with different amounts of H_2_O_2_ followed by the addition of a given amount of AuNPs. 

In Mixt.1, 4-MPBA molecules are firstly adsorbed on the surface of AuNPs as thiolate by losing its proton, and then H_2_O_2_ reacts with the immobilized 4-MPBA molecules. As displayed in Figure 3A, with the increase of H_2_O_2_ in the 4-MPBA/AuNP solution, besides the almost constant peak around 523 nm, a new absorbance peak gradually appears between 600 to 900 nm, and the solution color visibly changes from ruby red to blue. These results suggest that the aggregation of AuNPs enhances with the increase of H_2_O_2_, which is further proved by TEM images (see Figure S2).

**Figure 4:**
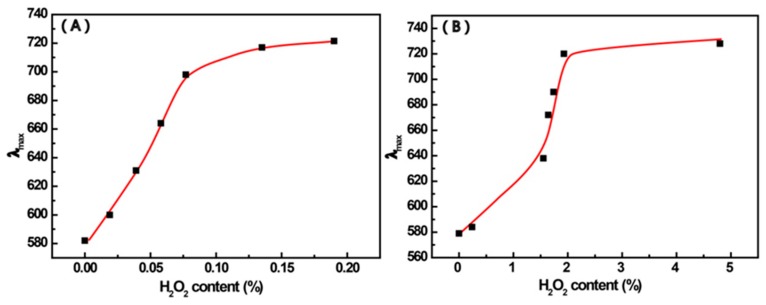
The change in the wavelength of maximum absorbance with the content of H_2_O_2_ in Mixt.1 (**A**) and Mixt. 2. (**B**).

Correspondingly, the change of the wavelength at maximum absorbance with the concentration of H_2_O_2_ is shown in Figure 4. In Figure 4A, the λ_max_ increases linearly with H_2_O_2_ concentrations in the range of 0~0.025%. The color change of Mixt.1 could be understood with the help of the following explanations.

**Figure 5:**
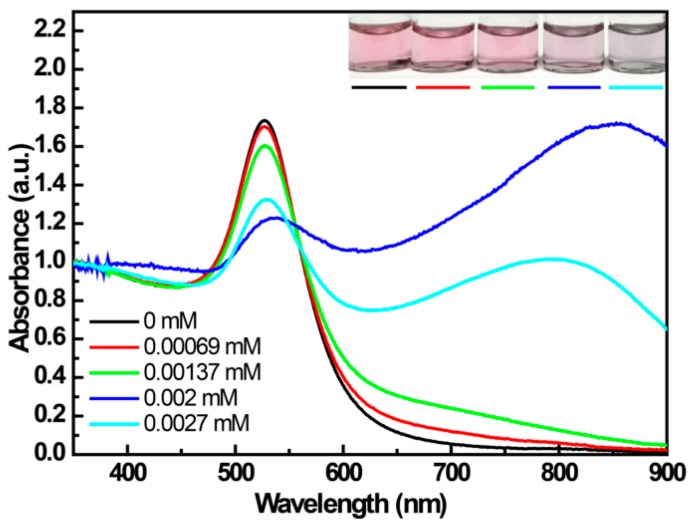
The change in the UV-Vis spectrum of Mixt.4 with 4-hydroxythiophenol (4-HTP) content.

It is well known that aryl boronate molecules can react selectively and efficiently with H_2_O_2_ to yield its corresponding phenol form [16]. Here, in the presence of H_2_O_2_, 4-MPBA immobilized on AuNPs is oxidized into 4-hydroxythiophenol (4-HTP), and this change is responsible for the aggregation of 4-MPBA/AuNPs. To verify this extrapolation, in the UV-Vis spectra of Mixt.4, the change in the UV-Vis spectra of the AuNP solution in the presence of different amounts of 4-HTP was recorded (Figure 5). Compared with 4-MBPA (Figure 1), even under the condition where the concentration of 4-HTP is much less than 4-MBPA, a particularly obvious absorbance peak appears above 600 nm, and the solution color clearly changes from ruby red to blue. This result confirms that the transition of 4-MPBA/AuNPs into 4-HTP/AuNPs is favorable to the aggregation of AuNPs. This inclination is actually related to the electrostatic interaction. The pKa of 4-MPBA (9.2) [21] is lower than that of 4-HTP (about 10) [30]. Namely, the formation of dissociated 4-MPBA with a negative charge is easier than that of 4-HTP. The results from Zeta-potential determination confirmed the change of the charge density for Mixt.1. As shown in Figure 2, the absolute value of the Zeta-potential decreases for the mixture of 4-MPBA (0.16mM)/AuNPs with the increase of H_2_O_2_ (without H_2_O_2_, −31.8 eV; 0.019% H_2_O_2_,−31.3 eV; 0.058% H_2_O_2_, −28.9 eV; 0.19% H_2_O_2_, −25.2 eV). Apart from the decrease in charge density, hydrogen bonding is likely another factor in enhancing the interaction between 4-HT and AuNPs. The phenol groups are expected to be partially dissociated at around pH 10. The hydrogen bonding between the dissociated phenolate group and the undissociated phenol group is stronger than that between undissociated phenols [31].

For Mixt.2, 4-MPBA was first mixed with H_2_O_2_ in a lower ratio of 4-MPBA to H_2_O_2_, and then a given amount of AuNPs was separately added. In these mixtures, 4-MPBA molecules are first free and oxidized by H_2_O_2_, and then the products interact with AuNPs. The UV-Vis spectra of Mixt.2 are shown in Figure 3B, the change of the wavelength at maximum absorbance with the concentration of H_2_O_2_ is shown in Figure 4B. Like Mixt.1, besides the almost unchanged peak at around 523 nm, a new peak between 600 to 900 nm continuously enhances with the increase of H_2_O_2_, and the solution color visibly changes from ruby red to blue. However, compared with Mixt.1, the concentration of H_2_O_2_ that induces the color change of solution is remarkably higher in Mixt.2. This difference could also be seen in the comparison of Figure 4A,B. For example, the color change caused by 0.058% H_2_O_2_ in Mixt.1 is similar to that caused by 1.64% H_2_O_2_ in Mixt.2. The obvious color change of Mixt2is with aH_2_O_2_ concentration in the range of 0~1.93%. In principle, this concentration possesses relativity, because it depends on the ration of AuNPs to 4-MPBA. Here, this concentration is only to show the effect of H_2_O_2_ concentration on the trend in the color change of the mixture (AuNPs-4-MPBA-H_2_O_2_). However, for Mixt.1, the corresponding H_2_O_2_ concentration is in the range of 0~0.025%. This difference is explained in the next section. 

### 3.3. The Products Formed in the Reaction between 4-MPBA and H_2_O_2_

Although many colorimetric probes based on the aggregation of AuNPs involving 4-MPBA and H_2_O_2_ have been reported, to our knowledge, it has rarely been reported with systematical characterization of products formed in the reaction. Here, the main products formed in the reaction between 4-MPBA and H_2_O_2_ were characterized.

**Figure 6:**
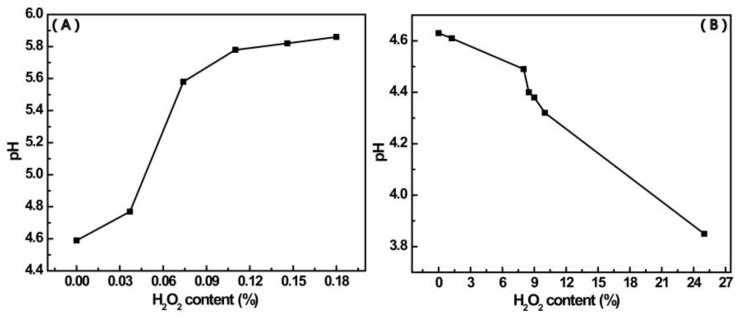
The change in pH of the mixtures containing a different ratio of 4-MPBA to H_2_O_2_ with the content of H_2_O_2_. (**A**) A higher ratio of 4-MPBA to H_2_O_2_ (1.87 mM 4-MPBA, 0~0.18% H_2_O_2_); (**B**) a lower ratio of 4-MPBA to H_2_O_2_ (0.58 mM 4-MPBA, 0~25% H_2_O_2_).

Figure 6 shows the change in pH with the content of H_2_O_2_ for the two mixtures that contain different ratios of 4-MPBA to H_2_O_2_ in the absence of AuNPs. For the mixture with the higher ratio of 4-MPBA to H_2_O_2_ (as similar to Mixt.1)_,_ the pH gradually increases with the increase of H_2_O_2_ content (Figure 6A). In contrast, for the mixture with the lower ratio of4-MPBA to H_2_O_2_ (as similar to Mixt.2_,_ the pH decreases with the increase of H_2_O_2_ (Figure 6B). For the former mixture, H_2_O_2_ oxidizes 4-MPBA (pKa = 9.2) to form 4-HTP (pKa = 10) and boric acid (pKa = 8.9) [32]. Although the formation of boric acid leads toa decrease of pH due to its relatively lower pKa, the formation of 4-HTP leads to an increase of pH due to 4-HTP having a higher pKa. In this situation, H_2_O_2_ is not enough to continue the reaction because of the stoichiometric coefficient for the reaction of 4-MPBA with H_2_O_2_. Thus, for the mixture with a higher ratio of 4-MPBA to H_2_O_2_, the pH of the solution increases with H_2_O_2_ content. However, for the mixture with the lower ratio of 4-MPBA to H_2_O_2_ (Figure 6B), besides the aforementioned reaction and due to excess H_2_O_2_, 4-HTP further is oxidized by H_2_O_2_ to form bis(4-hydroxyphenyl) disulfide (BHPD) (pKa = 10) [33,34], and the formed boric acid reacts with excess H_2_O_2_ to form peroxoboric acid ((HO)_2_BOOH) with a stronger acidity (pKa = 5.7) [32,35]. Although the formation of 4-HTP and BHPD leads to an increase of pH due to their higher pKa, the formation of BHPD involves proton consumption, and peroxoboric acid has a stronger acidity. As a result, not only is the process of pH increase masked but the pH also presents as a downtrend.

**Figure 7:**
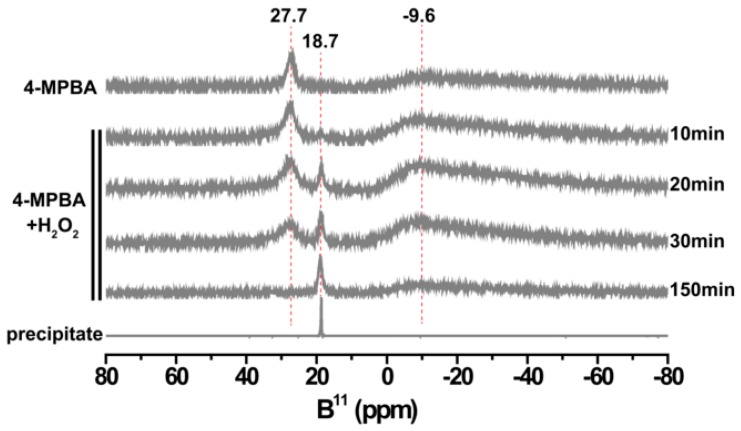
The ^11^B NMR spectra for different samples. Top: 4-MPBA; middle: the change in the spectrum of the mixture of 4-MPBA and H_2_O_2_ (0.15 M 4-MPBA, 0.94 M H_2_O_2_) with time; bottom: white precipitate formed during the reaction of 4-MPBA and H_2_O_2_.

The formation of boronic acid and BHPD in the mixture containing excess of H_2_O_2_ was testified using ^11^B NMR spectroscopy (Figure 7). From Figure 7, the following results in the spectra are found. The peak at 27.7 ppm that is attributed to 4-MPBA appears [36], and this peak gradually weakens with time and finally disappears entirely, while a new characteristic peak at 18.7 ppm assigned to B(OH)_3_ appears and progressively strengthens [37]. Additionally, the broader peak centered at −9.6 ppm that originates from (HO)_3_BOOH^−1^ or (HO)_2_B(OOH)_2_^−1^ gradually increases and then decreases [32]. This result proves the conjecture on the formation of the peroxoboric acid species. As for the decrease of peroxoboric acid species with time, it can be interpreted in terms of the decrease of their content with the decrease of pH [32].

**Figure 8:**
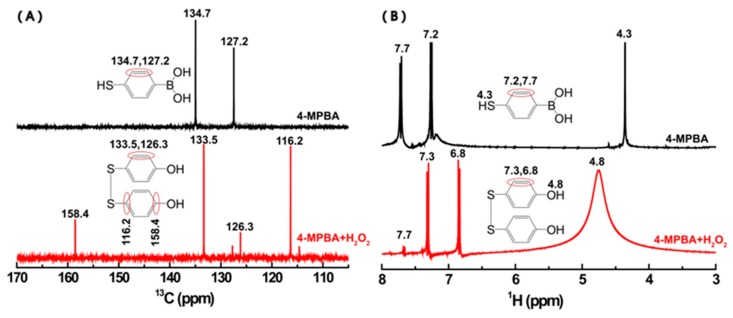
The ^13^C (**A**) and ^1^H (**B**) spectra for the reaction solution of 4-MPBA and H_2_O_2_ (0.15 M 4-MPBA, 0.94 M H_2_O_2_) in the late reaction period.

More importantly, the formation of BHPD was also testified by using ^13^C and ^1^H NMR spectroscopy. Figure 8 shows the ^13^C and ^1^H spectra of the reaction solution of 4-MPBA and H_2_O_2_ in the late reaction period. For the mixture of H_2_O_2_ and 4-MPBA, two peaks at 127.2 ppm and 134.7 ppm attributed to the primary carbon of benzene ring of 4-MPBA decreases [38], while the characteristic peaks of BHPD appears at 116.2 ppm, 126.3 ppm, 133.5 ppm, and 158.4 ppm [39,40] (Figure 8A). 

These results are consistent with the finding shown in the corresponding ^1^H NMR spectra (Figure 8B). After the reaction between 4-MPBA and H_2_O_2_,the characteristic peaks (4.3 ppm, 7.2 ppm, 7.7 ppm) related to hydrogen atoms in 4-MPBA almost disappear to be replaced by the characteristic peaks (4.8 ppm, 6.8 ppm, 7.3 ppm) of BHPD [39,40]. The broadening of the peak centered at 4.8 ppm may be associated with an intermolecular exchange of proton between the phenolic hydroxyl andH_2_O [41]. It is worth noting that there is no peak to be observed beyond 8 ppm, suggesting that the condensation between 4-MPBA molecules did not occur [42].

**Figure 9:**
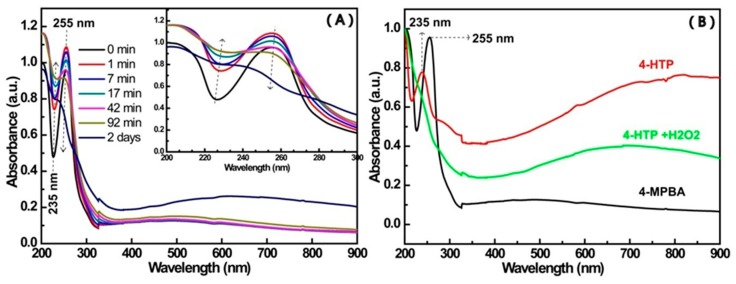
The change in the UV-Vis spectrum with time for different samples. (**A**) The change in the spectrum of Mixt.3 (0.15 mM MPBA, 0.012% H_2_O_2_) with time (inset: the spectra ranging from 200 nm to 300 nm); (**B**) 4-MPBA aqueous solution (black), 4-HTP aqueous solution (red), Mixt.5 (0.07 mM 4-HTP, 0.012% H_2_O_2_) after 20 min reaction (green).

The formation process of the products was also monitored by the following experiments. Figure 9A shows the change of the UV-Vis spectrum with time for the mixture of4-MPBA and H_2_O_2_ (Table 1, Mixt.3). Figure 9B presents the UV-Vis spectra of 4-MPBA, 4-HTP, and the product from the reaction of 4-HTP and H_2_O_2_ (Table 1, Mixt.5). Making a comparison between Figure 9A,B, the following results can be seen: The characteristic peak at 255 nm of 4-MPBA [43] (as shown in Figure 9B, black curve) constantly decreases with time, and the trough at 235 nm synchronously ascends. The gradual decrease of the peak at 255 nm indicates the continuous consumption of 4-MPBA, and the continuous raise of the trough at 235 nm, which is the exact location of the characteristic peak of 4-HTP (as shown in Figure 9B, red curve), suggests the continual formation of 4-HTP [44]. Furthermore, the peak profile of the product from 4-HTPand H_2_O_2_ (0.012%) (as shown in Figure 9B, green curve) is similar to that of BHPD [34] and that of the late reaction period of 4-MPBA and H_2_O_2_. 

All above results indicate that the reaction between 4-MPBA and H_2_O_2_ first produces 4-HTP and boric acid followed by the formation of BHPD. Moreover, in the case of excess H_2_O_2_, peroxoboric acid can form. Although alkane thiols [45,46,47] and aryl thiols [34,48,49,50] oxidized by H_2_O_2_ to form the corresponding disulfide have been widely reported, a similar reaction for 4-MPBA as one for aryl thiols has rarely been reported. Khutoryanskiy et al. only employed the reaction of 4-MPBA oxidized by H_2_O_2_ to form disulfide bonds for cross-linking PVA (polyvinyl alcohol) [51]; however, the formation of boric acid through breaking the C-B bound was not mentioned, and the formation of disulfide bond was not characterized.

**Figure 10:**
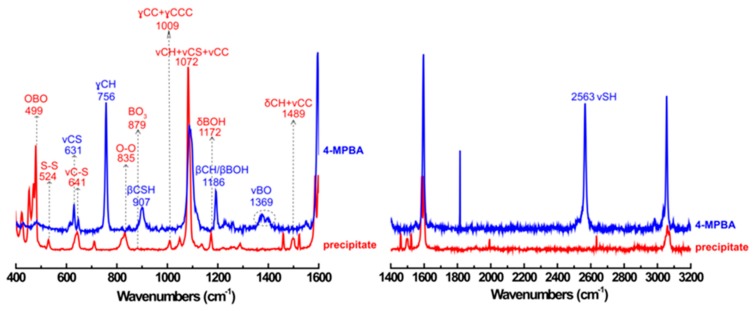
The Raman spectrum of white precipitate formed during the reaction of 4-MPBA (1.87 mM) and H_2_O_2_ (0.08%).

Interestingly, an insoluble white precipitate was observed during the reaction of 4-MPBA and H_2_O_2_ (see Figure S3A). Raman spectroscopy was used to identify the components of the precipitate. As shown in Figure 10, the Raman spectrum of 4-MPBA is well coincided with that in literature [52]. For the white precipitate, the peaks assigned to BHPD are found at 1489 cm^−1^ (ν_CC_ + δ_CH_), 1009 cm^−1^ (γcc + γccc), 641 cm^−1^(ν_C–S_) [53], 1072 cm^−1^ (ν_CH_ + ν_CS_ + ν_CC_) [54], 524 cm^−1^(ν_S–S_), and 499 cm^−1^(ν_OBO_) [55], being accompanied with the disappearance of the peaks attributed to 4-MPBA at 2563 cm^−1^ (ν_SH_), 1369 cm^−1^ (ν_BO_),1186 cm^−1^ (β_CH_ + β_BOH_), 907 cm^−1^ (β_CSH_), 756 cm^−1^ (γ_CH_), and 631 cm^−1^ (ν_CS_) [52]. These results confirm again that the reaction between 4-MPBA and H_2_O_2_ can form 4-HTP and BHPD. This conclusion was also proven by the XPS result shown in Figure S3B. The characteristic peak at 164.7 eV for the S-S bond in BHPD appears beside the peak at 163.57 eV, which is attributed to the S-H of 4-HTP [56]. Furthermore, it can be found that the bands assigned to boronic acid emerge at 1172 cm^−1^ (δ_BOH_), 879 cm^−1^ (ν_S(BO3)_), and 499 cm^−1^ (δ_OBO_) [57,58], and the band at 835 cm^−1^ is confidently assigned to the stretching mode of the peroxide bond (O-O) [59,60], indicating that theperoxoboric acid species exists in the white precipitate. The results from the analysis of the white precipitate are in agreement with the conclusion deduced from other data mentioned above.

### 3.4. The Aggregation of AuNPs Caused by the Interaction of 4-MPBA and H_2_O_2_

As shown in Figure 3, with the increase of the concentration of H_2_O_2_, regardless of the 4-MPBA firstly being mixed with AuNPs (Figure 3A) or H_2_O_2_ (Figure 3B), the new peak ranging from 600 to 900 nm in the UV-Vis spectra obviously increases and red-shifts. However, for two mixtures, the demanded amount of H_2_O_2_ that results in their color change has an evident discrepancy. In view of the aforementioned features of the reaction of 4-MPBA and H_2_O_2_, the differences between the UV-Vis spectra forMixt.1 and Mixt.2as shown in Figure 3 can be understood. 

According to the conclusions mentioned above, the condensation between 4-MPBA molecules was not found. In reality, the condensation between aryl boric acid should be carried out under heating conditions [40,41]. In fact, Figure S1 Eindicates that the aggregation of 4-MPBA/AuNPs is not found with pH values ranging from 4 to 10 as adjusted by HCl and NaOH, implying that the reaction of the boric acid moiety of 4-MPBA with citric acid almost does not happen in this situation. The obvious aggregation was observed when PBS was used to adjust pH (Figure S1A–D). The results from Figure S1 indicate that aggregation of 4-MPBA/AuNPs induced by pH changes is attributed to a decrease in electrostatic action rather than condensation. James et al. also reported that the species formed through the reaction between citrate and aryl boric acidis scarcely present in their mixture with a pH greater than 6 [61]. On the basis of our research and reported literature, we believe that the aggregation of citric acid-capped AuNPs in Mixt.2 possibly originates from the change in electrostatic interaction. The result shown in Figure 2 confirms this conclusion. 

**Figure 11:**
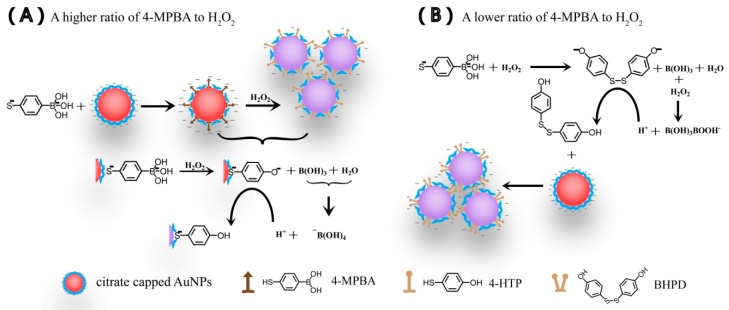
The proposed possible mechanisms for the aggregation of citrate-capped AuNPs. (**A**) Mixture ((AuNPs + 4-MPBA) + H_2_O_2_) with a higher ratio of 4-MPBA to H_2_O_2_; (**B**) mixture ((4-MPBA + H_2_O_2_) + AuNPs) with a lower ratio of 4-MPBA to H_2_O_2_.

Upon the basis of our investigative findings, we propose the possible mechanisms for the aggregation of AuNPs caused by the interaction of 4-MPBA and H_2_O_2_ in Mixt.1 and Mixt.2. As shown in Figure 11A, for Mixt.1 with the higher ratio of 4-MPBA to H_2_O_2_, 4-MPBA partly replaces citric acid on AuNPs, and then H_2_O_2_ reacts with the boric acid moiety of the immobilized 4-MPBA to form 4-HTP/AuNPs and boric acid. Citric acid with three negative charges is replaced by 4-HTP with one loaded negative charge, resulting in the aggregation of AuNPs, and the hydrogen bond between 4-HTP strengthens the aggregation of AuNPs. For Mixt.2 with the lower ratio of 4-MPBA to H_2_O_2_ as shown in Figure 11B, H_2_O_2_ reacts with free 4-MPBA to form BHPD and boric acid, and then BHPD molecules are immobilized onto the surface of AuNPs. Compared with 4-HTP, the immobilization of BHPD onto the surface of AuNPs is more difficult due to BHPD having greater steric hindrance (as shown in Figure 11A,B). Consequently, only when the concentration of BHPD is higher than that of 4-HTP can AuNPs modified by them have the same charge density. In consideration of the consumption of H_2_O_2_ in the formation of BHPD, the minimum concentration of H_2_O_2_ that results in the aggregation of AuNPs in Mixt.2 is naturally higher than that in Mixt.1. In addition, Mixt.2 contains relatively high amounts of H_2_O_2_, and thus the formation of peroxoboric acid with strong acidity is initiated. In such an event, the protonation of BHPD is enhanced.

## 4. Conclusions

The difference in the aggregation of AuNPs occurring in the mixture of ((4-MPBA + AuNPs) + H_2_O_2_) and the mixture of ((H_2_O_2_ + 4-MPBA) + AuNPs)has been found for the first time, and the corresponding mechanisms were investigated. Based on the experimental results, the following conclusions can be drawn:

(1) Compared with the mixture of ((H_2_O_2_ + 4-MPBA) + AuNPs), the color change of the mixture of ((4-MPBA + AuNPs) + H_2_O_2_) is more sensitive to H_2_O_2_, and this diversity that stems from the reaction of free and immobilized 4-MPBA with H_2_O_2_ is different. For the mixture of ((H_2_O_2_ + 4-MPBA) + AuNPs), free 4-MPBAis oxidized by H_2_O_2_ to form BHPD with greater steric hindrance and peroxoboric acid with stronger acidity. However, for the mixture of ((4-MPBA + AuNPs) + H_2_O_2_), the immobilized 4-MPBAis oxidized by H_2_O_2_ to form 4-HPT and boric acid. These differences make the mixture of ((4-MPBA + AuNPs) + H_2_O_2_) more sensitive to the aggregation of AuNPs caused by H_2_O_2_ than the mixture of ((4-MPBA + H_2_O_2_) + AuNPs).

(2) The aggregation of the citrate-capped AuNPs in the two mixtures mainly stems from the change in the charge density on the surface of AuNPs. 

(3) The formation of boric acid or peroxoboric acid in the reaction between 4-MPBA and H_2_O_2_ can affect the pH of the medium. The effect of this pH change on the aggregation of AuNPs should not be ignored. 

These findings not only offer a new strategy in colorimetric assays to respectively meet sensitivity and broad ranges of H_2_O_2_ detection but also assist in deepening the understanding of the aggregation of citrate-capped AuNPs involved in 4-MPBA and H_2_O_2_. Additionally, based on our new results, some possible methods in the determination of specific target analytes will be developed. For example, the detection of hydrogen peroxide produced from the reaction of glucose oxidase and glucose can be used to determine glucose; melamine could be determined based on the fact that the consumption of melamine combined with H_2_O_2_ could decrease the concentration of H_2_O_2_ and cause a change in the aggregation of 4-MPBA/AuNPs. In principle, the 4-MPBA/AuNPs system could be used to determine some specific substances that are related to the formation or consumption of H_2_O_2_.

## Figures and Tables

**Table 1:** Reaction conditions involved in the investigation.

Reactants	Mixt.1 ^a^	Mixt.2 ^a^	Mixt.3	Mixt.4 ^b,d^	Mixt.5
4-MPBA (mM)	0.16	1.28	0.15	---	---
4-HTP (mM)	---	---	---	0_~_0.0027	0.07
H_2_O_2_ (%)	0_~_0.19%	0_~_4.8%	0.012%	----	----
H_2_O_2_ (2 mL)	---	---	---	----	0.012%
AuNPs (3 × 10^12^ NPs/mL) ^c^	500 μL	500 μL	---	1 mL	---
Reaction time (min)	10	10	0_~_2 days	5	20

**Note:** Mixt.1: (4-MPBA + AuNPs) + H_2_O_2_; Mixt.2: (4-MPBA + H_2_O_2_) + AuNPs; Mixt.3: 4-MPBA + H_2_O_2_; Mixt.4: 4-HTP + AuNPs; Mixt.5: 4-HTP + H_2_O_2_. a: pH of AuNPs solution was adjusted to 10.8 by 0.5 M KOH. b: pH of AuNPs solution was 6.56. c: the size of AuNPs was ∼20 nm. d: the AuNP solution (3 × 10^12^ NPs/mL) was diluted 8 times.

## Supplementary Materials

The following are available online at https://www.mdpi.com/1996-1944/12/11/1802/s1, Figure S1: The change in the UV-Vis spectra of citrate-capped AuNPs modified by 4-MPBA (0.89 mM) with pH adjusted with different solutions. (A) 5 mM PBS; (B) 7.5 mM; (C) 10 mM; (D) 15 mM; (E) HCl and NaOH, Figure S2: The TEM images of citrate-capped AuNPs modified by 4-MPBA (0.16 mM) in the presence of different amount of H_2_O_2_. (1): 0; (2): 0.039%; (3): 0.058%; (4): 0.077%, Figure S3: The photo of the white precipitate formed during the reaction of 4-MPBA and H_2_O_2_ (A) and the XPS spectrum of S2p3/2 for the white precipitate (B).
