# Supplementary material for: Aggregation of Gold Nanoparticles Caused in Two Different Ways Involved in 4-Mercaptophenylboronic Acidand Hydrogen Peroxide
Source: Materials (Basel). 2019 Jun 3;12(11):1802. doi: 10.3390/ma12111802 (PMC6600739; doi:10.3390/ma12111802)
Supplement: Supplementary file 1 [file materials-12-01802-s001.pdf]

materials

Supplementary Material

Aggregation of Gold Nanoparticles Caused in Two Different Ways Involved in 4-Mercaptophenylboronic Acid and Hydrogen Peroxide

Runmei Li [1], Xuefan Gu [2], Xingtang Liang [1], Shi Hou [1] and Daodao Hu [1,*]

[1] Engineering Research Center of Historical and Cultural Heritage Protection, Ministry of Education, School of Materials Science and Engineering, Shaanxi Normal University, Xi'an 710062, China
[2] College of Chemistry and Chemical Engineering, Xi'an Shiyou University, Xi'an 710065, China
* Correspondence:Correspondence: daodaohu@snnu.edu.cn; Tel: 0086-29-81530717; Fax: 0086-29-81530702

Details in Characterization Techniques

UV-Vis Spectroscopy

A mixture (Mixt.1~Mixt.5 as shown in Table 1) solution (1 mL) was placed in a cuvette, and spectra were recorded within a wavelength range of 200–900 nm at room temperature. In the case of time-dependent measures, spectra of the reaction solution in a cuvette were recorded in situ.

Nuclear Magnetic Resonance Spectroscopy

In order to get a clear NMR signal for qualitative research, the amount of 4-MPBA was slightly higher (0.156M) than that used in Mixt.1 and Mixt.3 (0.15 mM), and it was dissolved in deuterated acetone followed by the addition of H_2O_2 (0.94M). Then the ^{11}B, ^{13}C, and ^{1}H spectra of the mixture were determined in situ with time.

Raman Spectroscopy

A laser power of 5 mW was incident on the sample in a 2 μm^2 diameter spot through a standard microscope objective lens. The Raman spectra were collected with a data point acquisition time of 90 s and a spectral range of 400–3200 cm^{-1}.

Transmission Electron Microscopy

20 uL of Mixt.1 with different concentrations of H_2O_2 was dropped onto the covered carbon membrane copper grids and then dried in air before observation. The corresponding TEM images of citrate-capped AuNPs modified by 4-MPBA in the presence of different amounts of H_2O_2 were obtained.

X-Ray Photo-Electron Spectroscopy

The pressure in the analysis chamber was greater than 5×10^{-9} Torr. To ensure the accuracy of the measured data, all binding energies were calibrated relative to the C 1s peak (284.6 eV) from hydrocarbons adsorbed onto the surface of the samples. In the investigation, the white precipitate formed during the reaction between 4-MPBA and H_2O_2 (Table 1 Mixt.2) was characterized by XPS.

Determination of pH Change

The pH changes of the solutions involved in Table 1 (Mix.1, Mix.2, Mix.3) were monitored by a PB-10 pH meter (Sartorius, Germany).

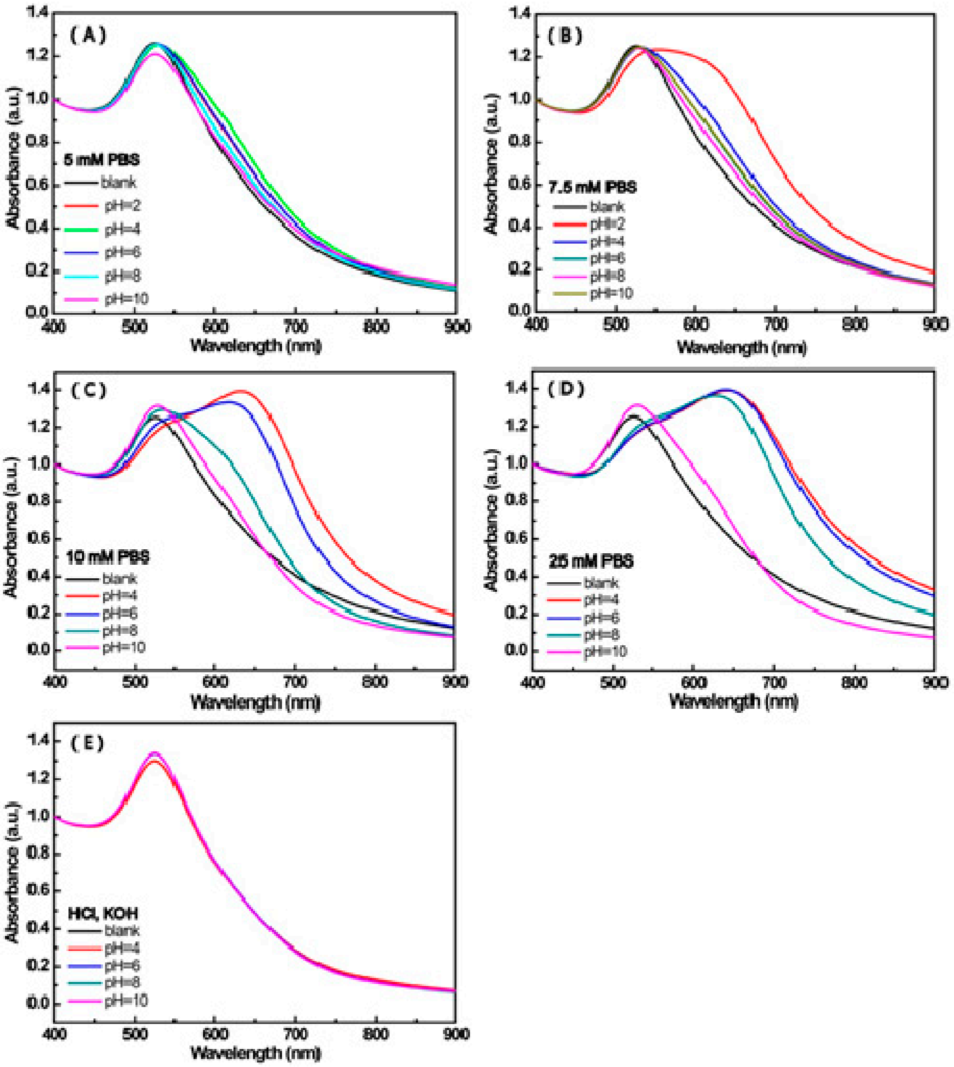

Figure S1. The change in the UV-Vis spectra of citrate-capped AuNPs modified by 4-MPBA (0.89 mM) with pH adjusted with different solutions. (**A**) 5 mM PBS; (**B**) 7.5 mM; (**C**) 10 mM; (**D**) 15 mM; (**E**) HCl and NaOH.

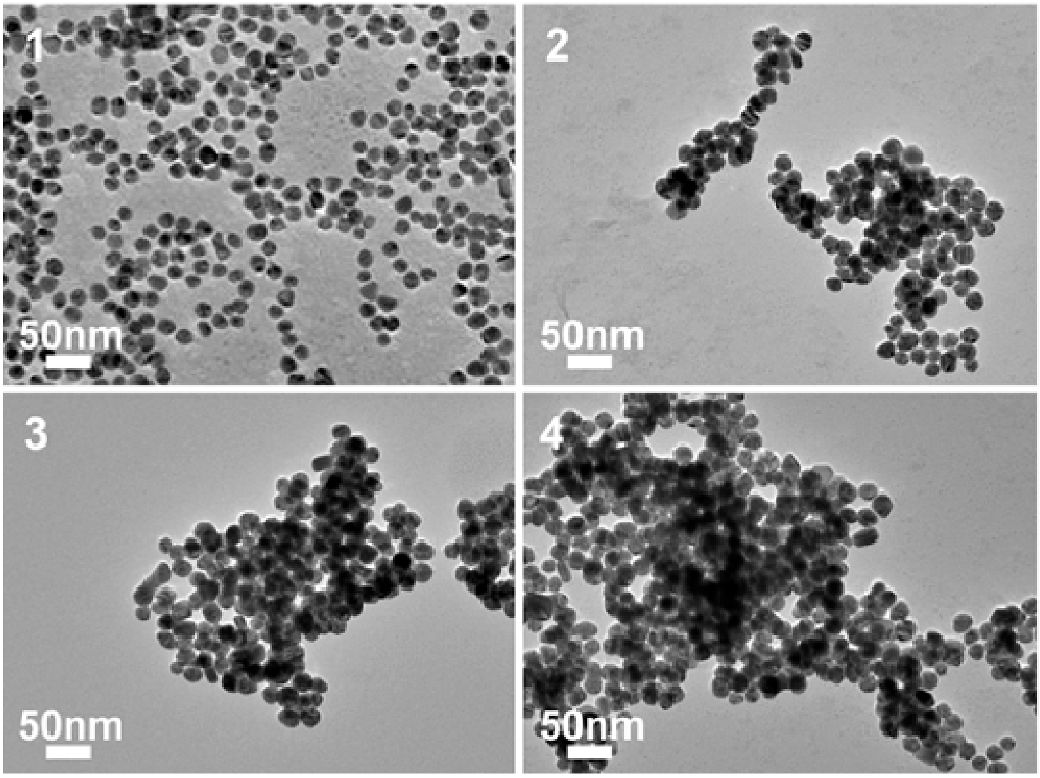

Figure S2. The TEM images of citrate-capped AuNPs modified by 4-MPBA (0.16 mM) in the presence of different amount of H_2O_2. (**1**): 0; (**2**): 0.039%; (**3**): 0.058%; (**4**): 0.077%.

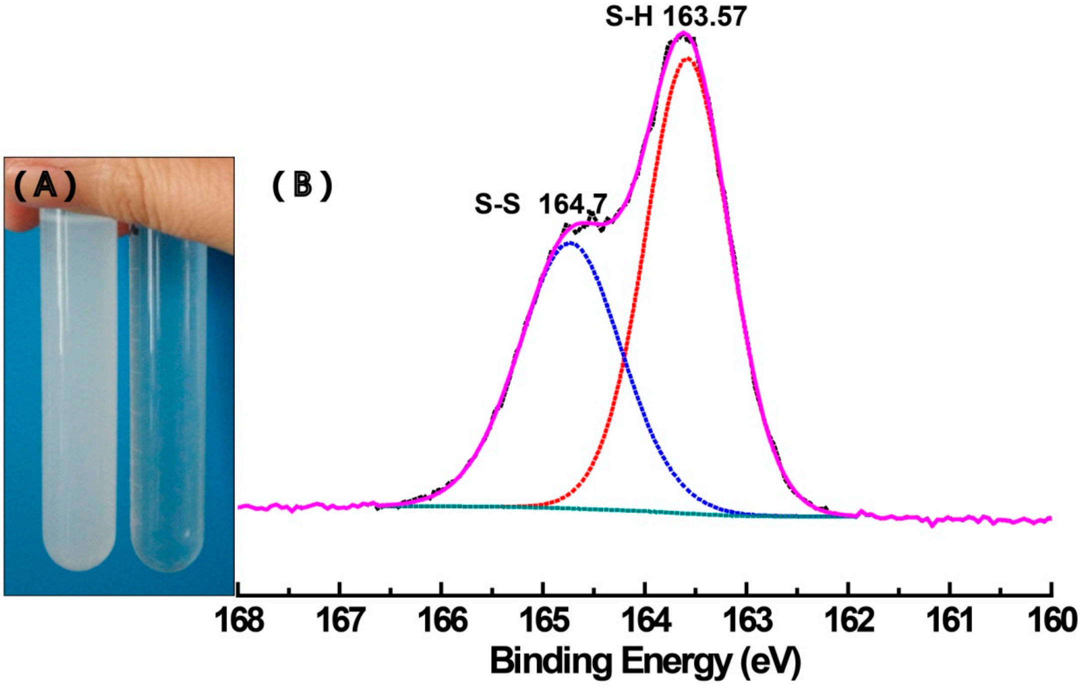

Figure S3. The photo of the white precipitate formed during the reaction of 4-MPBA and H_2O_2 (A) and the XPS spectrum of $S2p_{3/2}$ for the white precipitate (B).
